# The Treatment of Patients after Abdominal Operations

**Published:** 1895-10-05

**Authors:** Leonard A. Bidwell

**Affiliations:** Senior Assistant Surgeon to the West London Hospital, &c.


### Medical Progress and Hospital Clinics.

[*The Editor will be glad to receive offers of co-operation and contributions from members of the profession. All letters should be addressed to* The Editor, at the Office, 428, Strand, London, W.C.]

### THE TREATMENT OF PATIENTS AFTER ABDOMINAL OPERATIONS.

By Leonard A. Bidwell, F.R.C.S., Senior Assistant Surgeon to the West London Hospital, &c.

During the last few years a great change has come about in the treatment of abdominal cases, since it is only a short time ago, owing a good deal to the teaching of the special hospitals, that a sort of mystery was attached to the performance of such an operation as ovariotomy; thus, it was an invariable rule that every ovariotomy should be performed in a private room and not in the ordinary operating theatre, and that the case should be nursed afterwards in a special ward and by two special nurses. The operating-room was prepared by a steam spray, which was kept playing for two or three hours before the operation, and students admitted to the operation were submitted to the most stringent inquiries as to their having previously visited the post-mortem or dissecting rooms. Few, too, were admitted, owing to the smallness of the room. In fact, an ovariotomy was always considered to be totally different from any other surgical case, and as an operation of very great danger.

In my own practice all abdominal operations are performed in the ordinary operating theatre, since this is a more convenient place than a private room; the patients are removed, after the operation, to a general, medical, or surgical ward, where they are nursed by the ordinary ward nurses, and I do not recall a single hospital case where I have found it necessary to order special nurses.

With regard to the preparation of the patient for an abdominal operation, my own is very simple. The patient is kept in bed for two days, and on the second day before the operation two calomel and colycynth pills are given, followed by an enema on the morning before and on the morning of the operation. No purge is given on the evening preceding the operation, since the action of aperient medicine would prevent any chance of the patient having a good night's rest before the operation. One of my friends goes further than this, and considers that no purge should be given before a laparotomy, since the action of a purge weakens the patient and must set up a certain amount of irritation of the intestines. In his practice, an enema only is given on the day before the operation. On the other hand, some surgeons make much more extensive preparations. M. Lammelongue, for instance, keeps his patients in bed for a week before operation, and gives daily doses of $\beta$-naphthol in order to aseptisize the intestinal contents. He combines this treatment with free purging.

On the evening before the operation the patient should, when possible, have a hot bath, and the skin of the abdomen should be prepared in the following way: It is first thoroughly scrubbed with soft soap and water by a flesh brush, then rubbed with turpentine, and, lastly, covered with a compress soaked in a 1 in 20 solution of carbolic acid. In my opinion it is quite unnecessary and unjustifiable to insist upon shaving a patient before a laparotomy.

I prefer to do these operations in the morning so as to save the patient from the suspense of expectation and from the feeling of hunger, since on the day of the operation nothing but a little beef tea is given in the early morning. The patient should be warmly clad in flannel jacket and drawers.

These preparations apply to abdominal section for all conditions, but in gastro-enterostomies I have the stomach washed out with boric acid solution, and before hysterectomies I have the vagina washed out with 1 in 1,000 corrosive sublimate solution on the morning of the operation. In all pelvic operations, too, the catheter is passed by the nurse immediately before coming into the theatre. After the operation, which of course is carried out with every antiseptic precaution, the patient is put back in an ordinary bed which has been well warmed, with a pillow beneath her knees. As soon as the patient has come out of the anæsthetic she is allowed teaspoonfuls of hot water, but nothing else by the mouth for the first twelve hours. If she is violently sick a hand should be placed on the abdominal wound during the straining. The hot water is given even if the patient be sick, since I am sure that it is better that the stomach should have something to throw up than that the patient should go on retching ineffectually; the hot water also has the effect of washing out the mucus from the stomach. After twelve hours if there be no sickness the patient is fed in a precisely manner to any other severe surgical case; thus on the day after the operation a small cup of tea made with milk is given with drinks of barley water and beef tea at intervals, and custard pudding is allowed for dinner; on the third day fish or chicken is given according to the patient's inclination.

The old method of treating these cases by giving ice only to suck is bad, since the ice does not relieve the thirst so well as hot water, and has a paralysing effect on the intestines; hot water, on the other hand, aids in the setting up of peristaltic action. Personally I have not found that my patients suffer extremely from thirst when treated with hot water, and not allowed any morphia; some surgeons have found thirst very troublesome after operation, and the French surgeons recommend large enemas of salt and water as the best means of relieving it. I have had no experience of the treatment. The administration of morphia is responsible for producing thirst and vomiting in many cases. For this and other reasons I always withhold the drug.

The temperature and pulse should be noted by the nurse every four hours, and I am inclined to pay more attention to the pulse than to the temperature. If many adhesions have been separated it is not unusual to have a rise of temperature to 100 or 101 on the day following the operation, which is of no consequence; a rapid pulse, however, I consider more serious and regard it as indication for alcohol.

The use of nutrient enemata or suppositories is very seldom required; in fact, I only employ them when the patient has been in a low state before, and shows signs of exhaustion after the operation. As a general rule, I do not give any alcohol till the third day, when, if the patient desires it, she may have some champagne or brandy with her dinner; if, however, the pulse is rapid, I give brandy freely from the first.

There is one point in the after treatment of these cases to which I attach especial importance, and that is the management of the bowels in order to ensure a speedy re-establishment of peristaltic action after the paralysis of the gut, always produced to a greater or less degree by the exposure of the intestines in the wound. This point owes its importance to two conditions, one of immediate, and the other of more remote danger. The first condition is one known by the name of pseudo-ileus, and is an intestinal obstruction, brought about by paralysis of the gut from exposure or injury, followed by the formation of adhesions in such a position as to cause a kink. The trouble has often been confused with peritonitis, since its symptoms resemble that disease. There are, however, no post-mortem signs of inflammation. The resemblance of the symptoms of this condition to peritonitis has led a well-known gynæcologist to recommend the prevention and cure of peritonitis by saline aperients. Pseudo-ileus differs from ordinary obstruction in its rapidly fatal issue if unrelieved; the cause of this is not quite certain. According to the German theory, death is due to the migration of the bacillus coli commune from the paralysed gut into the peritoneal cavity, and to the absorption of toxines therefrom; according to other authorities the mischief is purely mechanical.

It is evident that the cause of this condition (namely paralysis of intestines) is at hand in nearly every case of laparotomy, and our indication of ensuring the re-establishment of the peristaltic action is the best, method of preventing this complication. To this end, I absolutely forbid the use of opium, either hypodermically or by the mouth, and endeavour to promote the passage of flatus from the rectum; it is the custom of my nurses always to pass a small rectal flatus tube within the anus six hours after the operation, and to continue its use every four hours until flatus is passed naturally. If no flatus is passed by the tube within forty-eight hours I do not waste time by making futile attempts to pass a long rectal tube, but immediately order a soap and water enema to be given; this nearly always produces an escape of flatus even if no fæcal matter comes away. Unless given for this purpose I do not usually give an enema until the evening of the third day, after which enemata or some mild aperient are given every other day. I attach the greatest importance to the frequent use of the flatus tube, and to the passage of flatus; in fact, I feel quite easy about a case when I have heard that she has passed flatus naturally. Another means of favouring the setting up of peristaltic action and the separation of recent adhesions is to change the patient's position in bed. On the day after the operation the patient may be turned from side to side, and on the third day she is allowed to sit up for a short time. The advantage of change of position on intestinal action is shown by a case related by a French surgeon, where a patient was suffering from pseudo-ileus, and no flatus escaped after enemata or aperients. She was in a very grave condition, and pulled herself up in bed to be sick; she felt something give inside, and soon after this the bowels acted, the patient making a complete recovery. In this case the obstruction was probably due to some slight adhesions, producing kinking, which were torn through by the weight of the bowel on her change of position. The third means, by which peristaltic movements are promoted, is the early and free feeding to which I have already alluded.

The more remote danger of delay in the re-establishment of peristaltic action is the formation of permanent adhesions or bands, which may produce acute or chronic obstruction some time afterwards. Several such cases have been recorded as occurring after ovariotomies, and it is not surprising that it should be so, when we consider that formerly in these cases the bowels were purposely kept at rest for about a week by opium and starvation, thus giving every opportunity for the formation of adhesions. Two such cases, one of acute, the other of chronic, intestinal obstruction, have recently been under my care in the hospital. The first patient was a young woman on whom ovariotomy had been performed, five months previously, by an obstetric physician at another hospital. Two hours before admission she was seized with violent abdominal pain, together with vomiting and collapse. I reopened the abdomen, and found two coils of intestine adherent to the abdominal scar, with a band passing between them; beneath this band another coil of ilium was strangulated. The band was divided, and the intestines were freed. Attention was paid to the early re-establishment of peristaltic action, and the patient made an uneventful recovery. The second patient was a woman, aged thirty-eight years, from whom the same physician had removed some sub-peritoneal fibroids and one ovary. Three years previously, eighteen months after the operation, the patient began to suffer from constipation and abdominal pain. For four months the pain had been severe and the constipation more difficult to overcome; she had also passed blood and mucus from the rectum. There was still a large fibroid to be felt. I reopened the abdomen and found the sigmoid flexure adherent to the fibroid, together with a number of bands passing from the great omentum to the growth. These were divided and the gut freed, the fibroid was then removed after the formation of flaps and the second ovary taken away. She made a rapid recovery and left the hospital quite well at the end of three weeks.

(*To be continued.*)

